# Measurement-Based Prediction of Inductive Ignition Risk Boundaries for Bridgewire Electro-Explosive Devices Under Transient Magnetic Fields

**DOI:** 10.3390/s26196345

**Published:** 2026-10-08

**Authors:** Hongyu Li, Yan Hu, Lizhi Wu, Yinghua Ye, Ruiqi Shen

**Affiliations:** 1 School of Chemistry and Chemical Engineering, Nanjing University of Science and Technology, Nanjing 210094, China; 323103010143@njust.edu.cn (H.L.);; 2 Micro-Nano Energetic Devices Key Laboratory, Ministry of Industry and Information Technology, Nanjing 210094, China

**Keywords:** transient magnetic field measurement, effective magnetic flux area, inductive coupling, bridgewire electro-explosive device, ignition risk boundary

## Abstract

Predicting the ignition risk of bridgewire electro-explosive devices (EEDs) under transient magnetic fields requires linking the local magnetic field history to the inductive response of the associated closed loop and the thermal response of the bridgewire–charge system. In this study, a measurement-grounded framework was developed by combining transient field measurement with a three-dimensional electromagnetic–thermal model. A custom inductive probe with a manufacturer-specified equivalent magnetic flux area of *A_p_* = 12,029 mm^2^ was used to obtain the local magnetic field rate history (*dB*/*dt*). In bench tests, the induced-voltage histories of closed loops with different measured areas were reconstructed from the probe signal using the loop-to-probe area ratio and used as inputs to the bridgewire–charge model. Three measured transient magnetic field waveforms were used to evaluate the predicted ignition boundaries. For Waveform I, the experimental and predicted critical-area intervals were (772, 824] and (804.2, 907.9] mm^2^, respectively. For Waveforms II and III, the experimental interval (542, 583] mm^2^ lay within the predicted interval (530.9, 615.8] mm^2^. The evaluated framework was further applied to the projectile-fixed magnetic field history of a five-stage coilgun, yielding a predicted critical equivalent loop radius interval of (14, 15] mm. The results demonstrate that inductive ignition risk depends on closed-loop magnetic flux coupling and the complete transient excitation history rather than on peak magnetic flux density or peak *dB*/*dt* alone. The proposed method establishes a quantitative link among local transient magnetic field measurement, installed loop magnetic flux coupling, and device-level ignition risk boundaries, providing a practical framework for electromagnetic safety assessment of projectile-borne EEDs under complex transient magnetic field environments.

## 1. Introduction

Electromagnetic launch systems convert pulsed electrical energy into projectile kinetic energy within a few milliseconds, making them attractive for high-speed launch applications [1,2]. Railguns rely on sliding electrical contact between the armature and rails, whereas multistage coilguns accelerate a projectile through the sequential discharge of multiple drive coils. This contactless acceleration mechanism avoids the sliding electrical contact inherent in railguns and, therefore, mitigates contact-related ablation, frictional wear, and arc erosion [3]. Research on coilguns and reluctance accelerators has focused primarily on launch performance, including launch efficiency, magnetic field arrangement, discharge circuit optimization, initial position matching, interstage triggering, pulse shaping, and velocity enhancement [4,5,6,7,8,9]. Recent studies have also addressed transient data analysis, projectile-state estimation, and novel launcher configurations [10,11,12]. By comparison, the electromagnetic environment inside the projectile has received less attention, despite the presence of electronic and electro-explosive components within modern projectiles. Studies on railguns have shown that in-bore magnetic flux density, projectile velocity, and current density evolution strongly influence the internal electromagnetic environment [13,14]. Research on projectile shielding, in-bore multiphysics responses, launch measurement, and magnetic field redistribution has further demonstrated that projectile-borne components are exposed to transient and spatially non-uniform electromagnetic conditions [15,16,17]. However, the transient magnetic field exposure experienced by projectile-borne components in a projectile-fixed frame during multistage coilgun launch remains insufficiently characterized.

Bridgewire electro-explosive devices (EEDs) are widely used for ignition and initiation in projectile-borne systems. Their response is governed by electrical energy deposition in the bridgewire, heat transfer to the energetic charge, and the resulting thermal evolution of the charge. Previous electromagnetic safety studies have focused predominantly on externally applied electromagnetic interference. Radio frequency irradiation and continuous-wave coupling experiments have been used to characterize the susceptibility and thermal response of bridgewire EEDs under external electromagnetic fields [18,19], while ignition temperature measurements and equivalent radiation test methods have been developed for high-power electromagnetic pulse environments [20,21]. Electromagnetic coupling at EED terminals has also been investigated in terms of lead wire current, input impedance, and equivalent antenna models [22,23]. These approaches provide useful tools for conventional susceptibility assessment; however, they mainly address field-to-lead or field-to-port coupling and do not directly characterize the magnetic flux coupling and induced electrical response of a closed conductive loop exposed to a transient, spatially non-uniform near-field environment.

In a multistage coilgun, the bridgewire, pins, connecting leads, and return path may together form an effective conductive loop. As the projectile moves through a transient and spatially non-uniform magnetic field, the magnetic flux linked with this loop varies continuously, inducing a pulsed voltage that may drive current through the bridgewire and produce unintended Joule heating. Classical studies of bridgewire ignition have shown that the response is governed by coupled electrical and thermal processes, including Joule energy deposition, thermal diffusion, bridgewire geometry, ignition energy, and charge reaction [24,25,26,27]. More recent studies of hot bridgewire EEDs have highlighted the importance of bridgewire–charge interface temperature and the temperature distribution within the charge [28,29,30]. Critical ignition temperature, pulse history, and coupled bridgewire–charge heat transfer further influence the predicted ignition threshold [31,32]. Therefore, external field strength, lead current, or input impedance alone cannot fully characterize ignition risk under coilgun launch conditions; the local launch field history and closed-loop geometry must also be considered.

Despite these advances, a quantitative relationship among transient magnetic field exposure, closed-loop inductive response, and the ignition behavior of projectile-borne EEDs remains insufficiently established. To address this issue, this study develops a measurement-based framework that integrates transient magnetic field characterization, closed-loop induced-voltage reconstruction, and bridgewire–charge electromagnetic–thermal prediction. The local magnetic field rate history is obtained using an inductive probe with a manufacturer-specified equivalent magnetic flux area, and the induced-voltage histories of loops with different enclosed areas are reconstructed from the measured probe signal under a geometric configuration consistent with the numerical model. Rather than relying on a single peak magnetic field indicator or an isolated electrical-response metric, the proposed framework preserves the complete transient excitation history and links the measured magnetic field exposure to the predicted ignition risk boundary. Three measured transient magnetic field waveforms are used to evaluate the predicted ignition boundaries, after which the framework is applied to the projectile-fixed magnetic field history of a five-stage coilgun to determine the critical equivalent loop radius interval under launch conditions.

## 2. Methodology

### 2.1. Multistage Coilgun Model and Projectile-Fixed Magnetic Field Extraction

**Figure 1:**
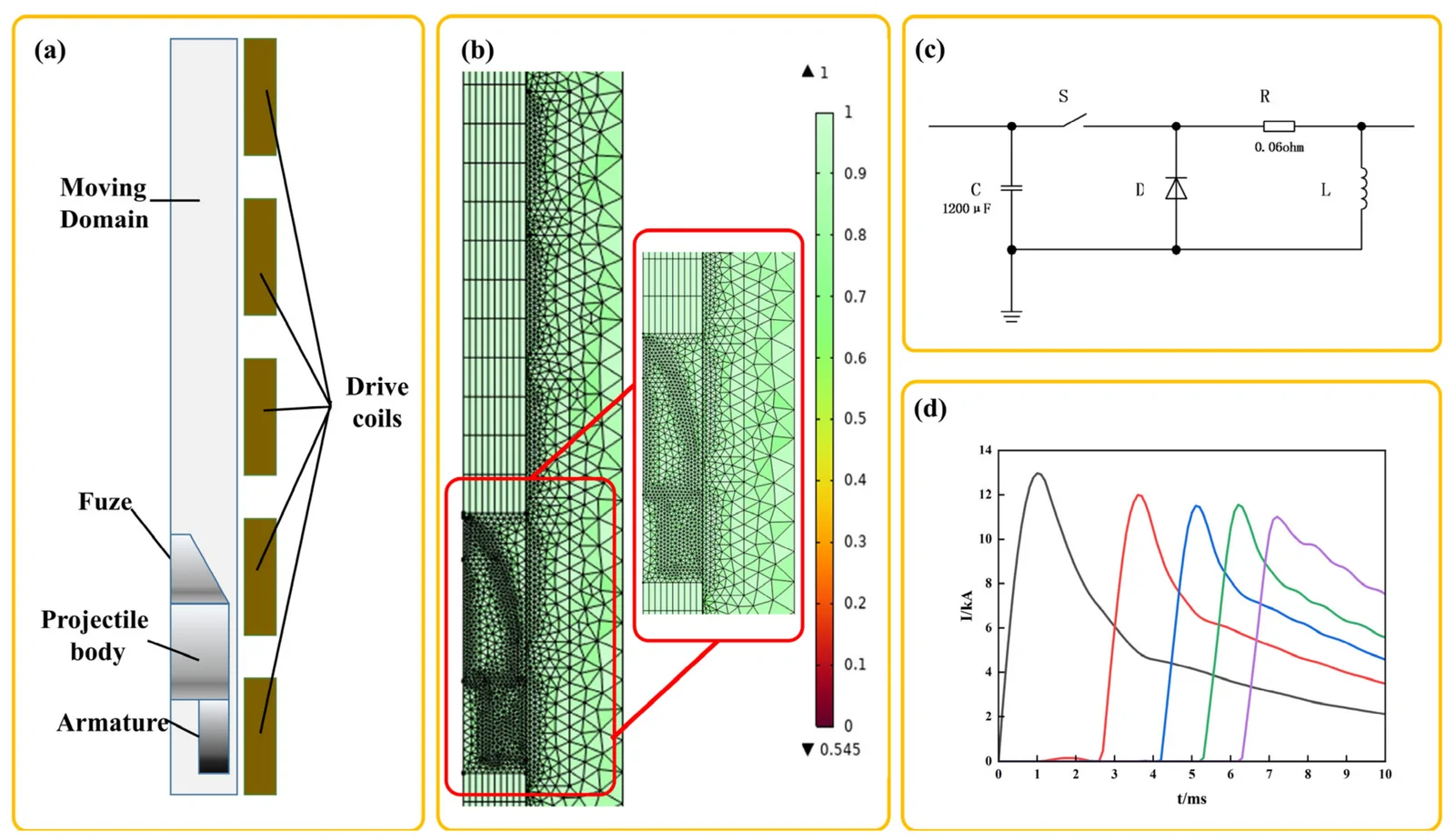
Multistage coilgun simulation model: (**a**) Two-dimensional axisymmetric physical model; (**b**) mesh quality distribution; (**c**) excitation circuit of the drive coils; (**d**) excitation current waveforms of the five drive coils.

The multistage coilgun model comprises five drive coils, a pulsed power supply circuit, a projectile, an armature, and a moving domain. To reduce computational cost while retaining the essential axial electromagnetic force and radial magnetic field characteristics during launch, a two-dimensional axisymmetric coupled electromagnetic–motion model was employed. The transient simulation geometry and the corresponding mesh are shown in Figure 1a,b.

Local mesh refinement was applied to the drive coils, armature, air gap, projectile, and other regions with steep electromagnetic field gradients. Most elements in the refined regions had element quality values close to unity. The armature was modeled as a non-rotating aluminum annulus, the drive coils as copper conductors, and the projectile as a representative structural alloy. The main geometric, electrical, and material parameters are listed in Table 1 [33].

To reproduce the dynamic in-bore launch process, the drive coils were supplied by an external pulsed power circuit, and each stage was triggered independently. The drive-coil excitation circuit is shown in Figure 1c. The energy storage capacitor had a capacitance of 1200 μF and an initial charging voltage of 8 kV. A position-dependent triggering strategy was adopted for all five stages, yielding the current waveforms shown in Figure 1d.

Previous studies [34] indicate that, for identical drive coils, the optimal trigger position shifts upstream as armature velocity increases. A drive coil may, therefore, need to be energized before the armature center reaches the coil center. A parametric study identified a center-to-center trigger offset of 0 mm for the first stage. For stages 2–5, maximum launch efficiency was achieved when the armature center was 18, 23, 32, and 43 mm behind the corresponding coil center, respectively.

The electromagnetic field was solved using the magnetic vector potential formulation, A, with the coil currents supplied by the external circuit. The magnetic flux density was obtained from the solved magnetic vector potential according to B = ∇ × A [35], where ∇ × denotes the curl operator. The axial motion of the projectile was governed by the electromagnetic force and an equivalent resistive force. The axial equation of motion is expressed as

(1) $$m\;\frac{d^{2}x}{{dt}^{2}}=F\left(x,t\right)-F_{r}$$

where *F*(*x*, *t*) is obtained by integrating the Lorentz force over the electromagnetic field domain, and *F_r_* includes gravity and the equivalent aerodynamic resistance.

As the EED moves together with the projectile, the local magnetic field at the installation region can be expressed as

(2) $$B_{launch}\left(t\right)=B_{launch}\left(t_{0}\right)+\int_{t_{0}}^{t}{\frac{\partial B}{\partial \tau }|}_{x=x\left(\tau \right),\;r=r_{0}}\;d\tau +\int_{t_{0}}^{t}v\left(\tau \right){\frac{\partial B}{\partial x}|}_{x=x\left(\tau \right),\;r=r_{0}}\;d\tau \;$$

where *B*_launch_(*t*_0_) denotes the magnetic flux density at the EED installation region at the reference time *t*_0_. The second term represents the accumulated contribution of the explicit temporal variation of the magnetic field along the EED trajectory, mainly arising from the transient drive coil currents. The third term represents the accumulated contribution of motion through the spatially non-uniform magnetic field, where *v*(*τ*) is the instantaneous projectile velocity and ∂*B*/∂*x* is the local axial magnetic field gradient. Both field derivatives are evaluated at the instantaneous EED position *x*(*τ*) and the fixed radial coordinate *r*_0_. Taking the total time derivative of Equation (2) yields

(3) $$\frac{dB_{launch}}{dt}=\frac{\partial B}{\partial t}+v\left(t\right)\frac{\partial B}{\partial x}$$

Equation (3) separates the rate of change in the projectile-fixed magnetic field into two components: the explicit temporal term, ∂*B*/∂*t*, arising from the transient discharge currents in the drive coils, and the motion-induced term, *v*(*t*)∂*B*/∂*x*, resulting from the projectile moving through a spatially non-uniform magnetic field. This decomposition distinguishes the contributions of the transient coil current excitation and projectile motion through the spatial field gradient to *dB*_launch_/*dt*.

Because *dB*_launch_/*dt* is obtained from the transient magnetic field history, its numerical evaluation is sensitive to temporal discretization. Preliminary calculations showed that relatively coarse time steps could lead to noticeable differences in the calculated magnetic field variation rate. Therefore, a time step refinement study was performed by successively halving the time step. The refinement was continued until the *dB*_launch_/*dt* waveform remained essentially unchanged over three consecutive refinements, indicating that the temporal discretization had reached a stable solution. Based on this convergence assessment, a final time step of 0.025 ms was adopted for the subsequent calculations. The resulting *B*_launch_(*t*) and dBlaunch/dt histories were then used as the excitation inputs for the EED electromagnetic–thermal model.

### 2.2. Closed-Loop Inductive-Coupling Model

**Figure 2:**
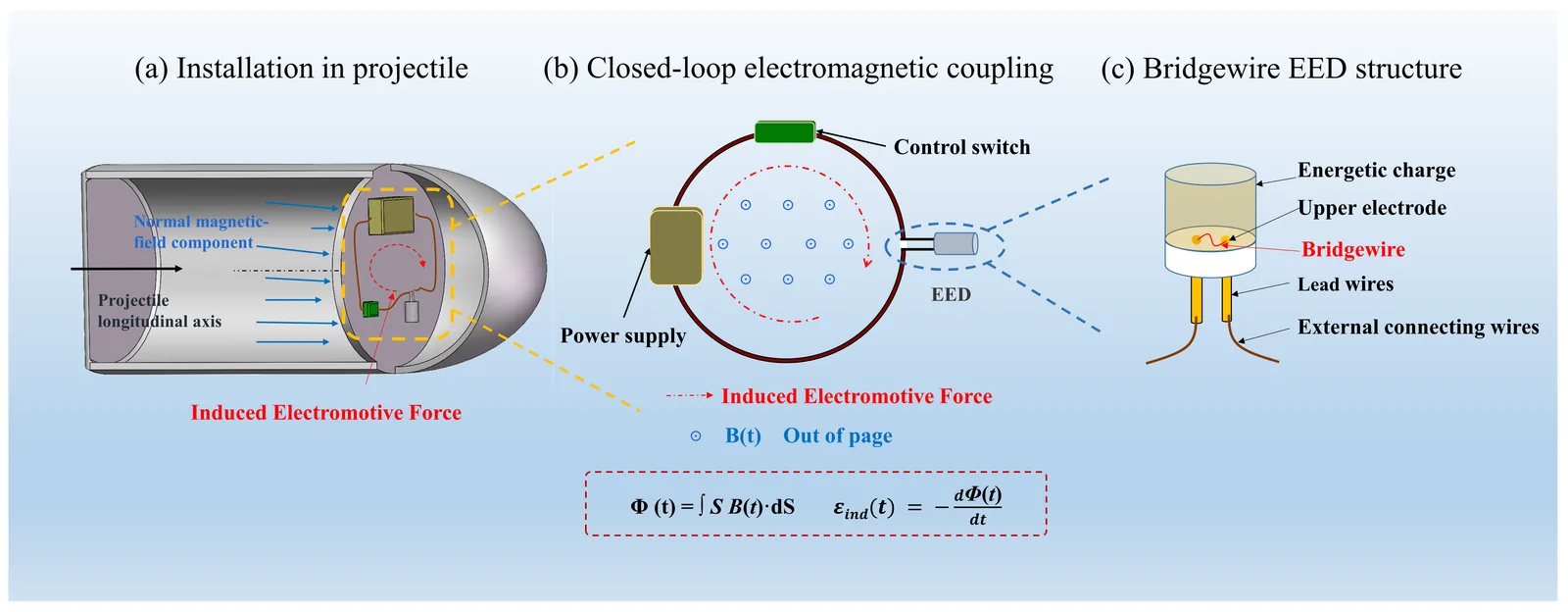
Schematic of closed-loop inductive coupling and bridgewire–energetic charge thermal response model.

A bridgewire EED is typically connected to its power supply through lead wires and external conductors. If these conductors form a closed loop inside the projectile, a time-varying magnetic flux linking the loop induces an electromotive force, as shown in Figure 2. For an input magnetic field *B_in_*(*t*), the induced loop voltage can be approximated as

(4) $$V_{ind}\left(t\right)=-A_{eff}\frac{dB_{in}\left(t\right)}{dt}$$

where *A*_eff_ is the effective magnetic flux area of the equivalent closed loop. For experimental validation, *B_in_*(*t*) = *B*_exp_ (*t*), with *B*_exp_ (*t*) representing the measured bench-top magnetic field waveform. For launch prediction, *B_in_*(*t*) = *B*_launch_(*t*), with *B*_launch_(*t*) representing the projectile-fixed magnetic field waveform obtained from the coilgun simulation.

In the transient model, the bridgewire current is obtained by coupling the equivalent circuit to the Electric Currents interface. For millisecond-scale magnetic field pulses, the current depends on the resistance of the loop conductors, the temperature-dependent bridgewire resistance, and the loop inductance. The effect of loop inductance was assessed by comparing the electromagnetic time constant of the loop with the magnetic field variation timescale. For the largest equivalent loop considered *R*_loop_ = 15 mm and a copper wire radius of 0.25 mm, the inductance is estimated to be approximately 79 nH using the single-turn circular loop approximation. The copper conductor has a resistance of approximately 8.1 mΩ, while the initial bridgewire resistance is approximately 1 Ω, resulting in a total initial resistance of about 1.01 Ω and an L/R time constant of approximately 0.078 μs. This electromagnetic time constant is several orders of magnitude shorter than the millisecond-scale magnetic field variation considered here. Joule heating further increases the bridgewire resistance and shortens the L/R time constant. The inductive contribution is, therefore, negligible over the relevant timescale, and a quasi-static resistive model is used for the electrothermal calculations.

### 2.3. Bridgewire–Charge Model

Bridgewire EED ignition involves Joule heating of the bridgewire, heat transfer to the surrounding energetic charge, exothermic decomposition of the charge, and thermal diffusion. Existing models commonly represent a small region near the bridgewire midpoint [34] and simplify the surrounding charge as a radially oriented cylindrical heat transfer domain. Ignition onset is then identified by the appearance of a local maximum in the radial temperature profile of the charge. To retain this thermal runaway mechanism while resolving the full geometry, a three-dimensional model of the bridgewire and surrounding charge is used.

**Figure 3:**
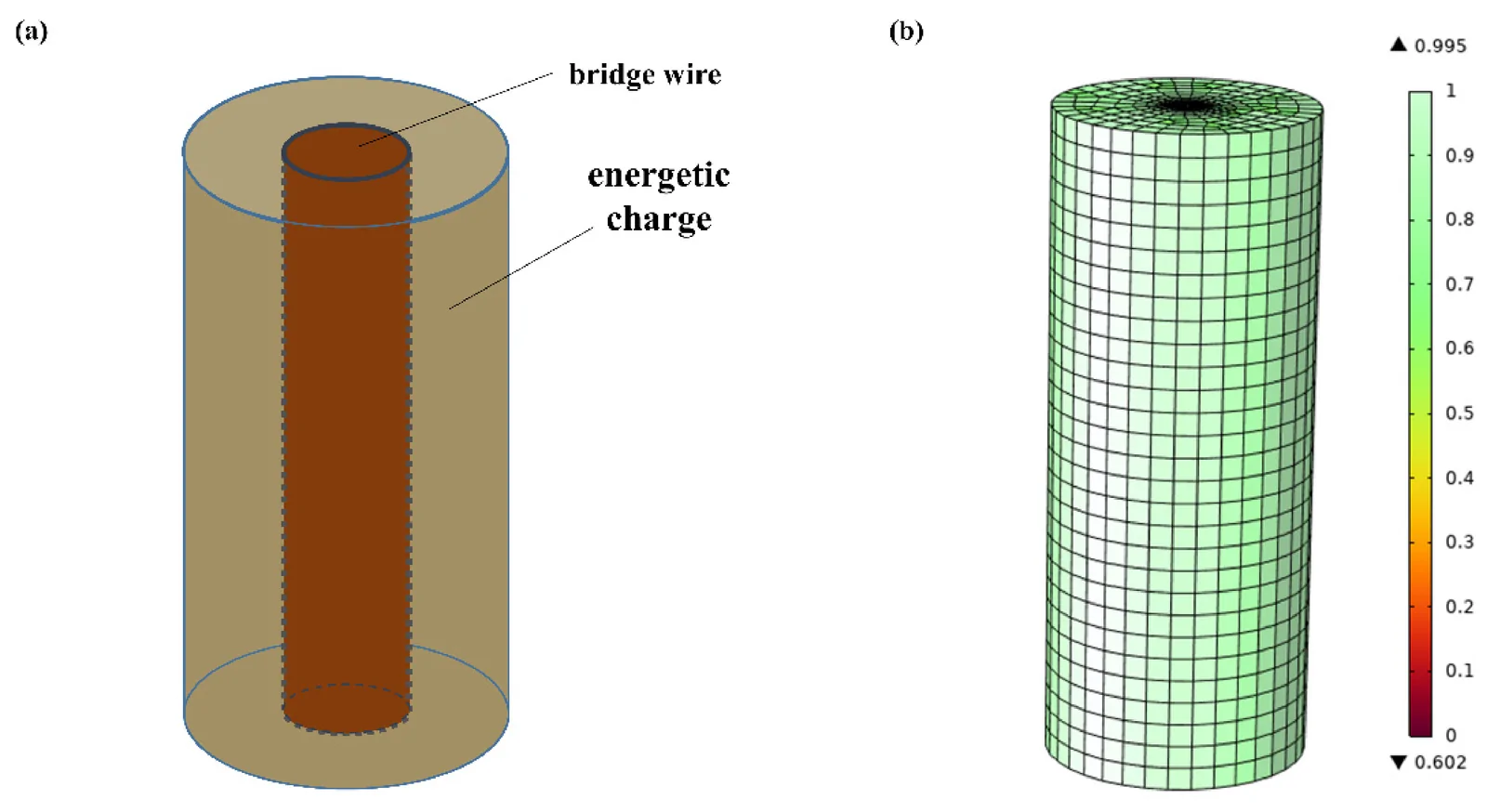
Bridgewire–charge model: (**a**) geometric model; (**b**) mesh distribution.

The bridgewire was modeled as a cylindrical conductor surrounded by a three-dimensional energetic charge domain. Figure 3a shows the geometry implemented in COMSOL Multiphysics 6.2 (COMSOL Inc., Burlington, MA, USA). The nominal bridgewire dimensions were taken from the technical specifications of the EEDs used in the experiments: a diameter of 20 μm and a free length of 1.2 mm. The detailed geometric parameters are listed in Table 2.

Figure 3b shows the mesh. Local refinement was applied around the bridgewire, with boundary-layer elements at the bridgewire–charge interface, where the thermal reaction is expected to initiate. Element quality exceeded 0.98 in the refined regions.

To retain the essential physics of the three-dimensional model while avoiding the large number of uncertain parameters required by a full structural model, the following assumptions were adopted.

First, the bridgewire and energetic charge are assumed to be in perfect thermal contact, so that temperature is continuous across the interface and interfacial thermal resistance is neglected. Second, Joule heating in the bridgewire is the only externally imposed heat source; no direct Joule-heating term is applied to the charge domain. Third, the energetic charge is treated as an isotropic equivalent solid with constant thermophysical properties before ignition. Fourth, thermal decomposition follows Arrhenius kinetics, with reactant consumption represented by an effective remaining reactant fraction. Fifth, the model is limited to heat accumulation up to the ignition criterion and does not describe post-ignition combustion propagation, gas generation, or structural failure.

### 2.4. Heat Transfer and Reaction Model of the Bridgewire–Charge System

The bridgewire heat source in the three-dimensional model is calculated using the Electric Currents interface. An induced voltage boundary and a ground boundary are applied at opposite ends of the bridgewire. The interface provides the current density *J* and electric field *E*, from which the volumetric Joule heat source is expressed as

(5) $$Q_{J}\left(x,t\right)=J\left(x,t\right)\cdot E\left(x,t\right)$$

Transient heat conduction is solved in both the bridgewire and energetic charge regions. In the bridgewire region, the temperature evolution is governed by Joule heating and heat transfer to the surrounding charge:

(6) $$\rho_{w}c_{w}\frac{\partial T_{w}\left(x,t\right)}{\partial t}=\nabla \cdot \left[k_{w}\nabla T_{w}\left(x,t\right)\right]+Q_{J}\left(x,t\right)$$

where *ρ_w_*, *c_w_*, and *k_w_* are the density, specific heat capacity, and thermal conductivity of the bridgewire, respectively.

In the energetic charge region, the temperature evolution is governed by three-dimensional heat conduction and heat release from thermal decomposition:

(7) $$\rho_{p}c_{p}\frac{\partial T_{p}\left(x,t\right)}{\partial t}=\nabla \cdot \left[k_{p}\nabla T_{p}\left(x,t\right)\right]+Q_{r}\left(x,t\right)$$

The heat release term of the energetic charge is described using an Arrhenius-type reaction model:

(8) $$Q_{r}\left(x,t\right)=\rho_{p}Qm\left(x,t\right)Aexp\left[-\frac{E_{a}}{R_{u}T_{p}\left(x,t\right)}\right]$$

where *ρ_p_*, *c_p_*, and *k_p_* are the density, specific heat capacity, and thermal conductivity of the energetic charge, respectively; *Q* is the heat of reaction per unit mass; *m*(*x*, *t*) is the effective remaining reactant fraction; *A* is the pre-exponential factor; *E_a_* is the activation energy; and *R_u_* is the universal gas constant.

The remaining reactant fraction decreases as thermal decomposition proceeds according to

(9) $$\frac{dm}{dt}=-mAexp\left[-\frac{E_{a}}{RT_{p}}\right]$$

The effective remaining reactant fraction *m*(*x*, *t*), which determines the heat release term in Equation (8), is obtained by solving the reaction-progress equation given in Equation (9). Therefore, Equation (9) describes the evolution of the reaction state, while Equation (8) calculates the corresponding heat release based on this evolving state.

The model is limited to the thermal response up to the ignition criterion. Post-ignition combustion propagation, gas generation, pressure evolution, and casing failure are not modeled.

### 2.5. Ignition Risk Criterion and Safety-Margin Index

The onset of thermal instability is identified by the emergence of a non-monotonic temperature profile along the direction normal to the bridgewire surface. When the charge is heated solely by the bridgewire and self-accelerating exothermic decomposition has not begun, its temperature decreases monotonically with increasing distance from the bridgewire. Once the local heat generation within the charge exceeds the heat dissipated by thermal conduction, an internal temperature maximum develops, indicating the onset of thermal instability.

In the three-dimensional model, the one-dimensional radial coordinate r is generalized to a local outward-normal coordinate s directed from the bridgewire surface into the charge. The charge temperature along the local normal direction is denoted by *T_p_*(*s*, *t*). The charge enters the ignition risk state when a positive temperature gradient first appears along the local normal direction:

(10) $${\frac{\partial T_{p}\left(s,t\right)}{\partial s}|}_{s=s_{e},\;t=t_{e}}>0$$

(11) $$T_{e}=T_{p}\left(s_{e},t_{e}\right)$$

where *t_e_* is the triggering time of the ignition risk criterion, se is the hot-spot location, and *T_e_* is the calculated temperature at the hot spot when the local temperature bulge is triggered.

Several sampling lines are arranged around the bridgewire from the bridgewire surface toward the interior of the charge. A discrete temperature bulge indicator is defined as

(12) $$G\left(t\right)={max}_{i}\left\{\frac{T_{p}\left(s_{i+1},t\right)-T_{p}\left(s_{i},t\right)}{s_{i+1}-s_{i}}\right\}$$

A small tolerance *G_tol_* is used during post-processing to suppress false triggering caused by numerical noise. When *G*(*t*) > *G_tol_*, the temperature profile along at least one local normal direction is no longer monotonically decreasing, and the ignition risk criterion is satisfied. The triggering time is defined as

(13) $$t_{e}=min\left\{t:G\left(t\right)>G_{tol}\right\}$$

This treatment avoids the empiricism associated with using a fixed interfacial temperature threshold alone to judge ignition. It also prevents the bridgewire thermal damage temperature from being incorrectly used as the ignition criterion of the energetic charge.

## 3. Experimental Validation and Critical Boundary Characteristics

To evaluate the model-predicted ignition boundaries under transient magnetic field excitation, firing tests were performed on bridgewire EEDs with different closed-loop areas under three experimentally measured magnetic field waveforms. The local magnetic-field variation rate history was obtained using an inductive probe with a known equivalent magnetic flux area. The loop planes were arranged perpendicular to the local magnetic field direction, consistent with the numerical model. For each loop area, the induced voltage history was reconstructed from the measured probe signal using the loop-to-probe area ratio. The experimental ignition/no-ignition boundaries were then compared with the model-predicted critical intervals.

The validation procedure comprised three stages. First, the local magnetic field variation rate history at the specimen location was measured using the inductive probe. Second, the probe signal was scaled by the measured loop-to-probe area ratio to reconstruct the voltage induced in each closed-loop configuration. Third, firing tests with progressively varying loop areas under the three measured waveforms established the experimental critical intervals. In the numerical model, the equivalent circular-loop radius was scanned in 1 mm increments for comparison with the experimental intervals.

### 3.1. Bench Test Magnetic Field System and Measurement Setup

**Figure 4:**
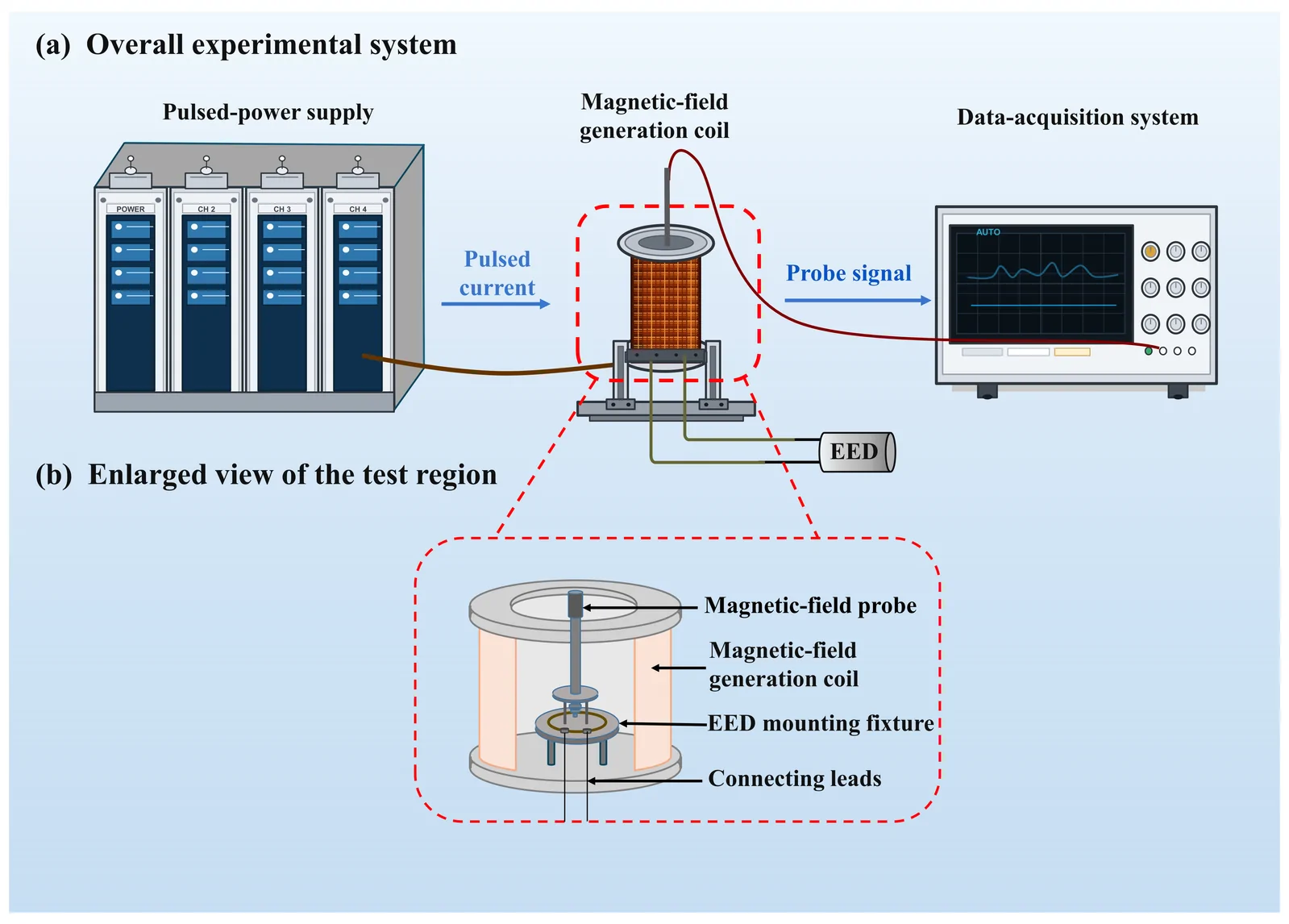
Schematic of the bench-top magnetic field experimental system and specimen arrangement.

The bench-top magnetic field system comprised a pulsed power supply, a field generation coil, a triggering and control unit, an EED fixture, closed-loop test configurations, a custom inductive magnetic field probe, and a digital storage oscilloscope, as shown in Figure 4. The pulsed power supply drove a millisecond-scale current through the field generation coil. The EED, test loop, and probe were placed within the same effective field region, with the loop plane perpendicular to the dominant local magnetic field direction to match the numerical model. Specimen position, loop orientation, probe position, and acquisition settings were held constant throughout the experiments.

The custom inductive probe was positioned adjacent to the specimen to characterize the local transient magnetic field. Its manufacturer-specified equivalent magnetic flux area, *A_p_* = 12,029 mm^2^, was used directly as the conversion coefficient. The probe output voltage was recorded with a digital storage oscilloscope (SDS2354X Plus) at a sampling rate of 2 MSa/s, a record length of 200 kpts, a time base of 10 ms/div, and an input impedance of 1 MΩ. The corresponding sampling interval of 0.5 μs was much shorter than the millisecond-scale variation timescale of the measured magnetic field waveforms.

### 3.2. Probe-Referenced Reconstruction of Loop-Induced Voltage

For the custom inductive magnetic field probe with a manufacturer-specified equivalent magnetic flux area of *A_p_* = 12,029 mm^2^, the probe output voltage can be expressed as

(14) $$u_{p}\left(t\right)=-A_{p}\frac{dB\left(t\right)}{dt}.$$

Therefore, the magnetic field variation rate in the specimen installation region can be reconstructed from the probe output as

(15) $$\frac{dB\left(t\right)}{dt}=-\frac{u_{p}\left(t\right)}{A_{p}}.$$

The magnetic flux density history, *B*_exp_(*t*), was then reconstructed by time integration.

During the firing tests, closed loops with different measured enclosed areas were positioned within the same effective magnetic field region as the probe. Their planes were perpendicular to the local magnetic field direction, so that the loop normal was approximately parallel to the field. Assuming that the field is approximately uniform over the relatively small loop area, the magnetic flux linked with the loop can be approximated as

(16) $$\Phi_{loop}\left(t\right)\approx A_{loop}B\left(t\right)$$

where *A_loop_* denotes the measured geometric area enclosed by the loop.

The corresponding induced voltage is, therefore

(17) $$u_{loop}\left(t\right)=-A_{loop}\frac{dB\left(t\right)}{dt}.$$

Substituting Equation (15) into Equation (17) gives

(18) $$u_{loop}\left(t\right)=\frac{A_{loop}}{A_{p}}u_{p}(t)$$

The induced voltage history for each loop was, therefore, reconstructed directly from the measured probe voltage waveform using the known area ratio *A_loop_*/*A_p_*_.

Under the controlled field alignment used in the bench tests, the effective magnetic flux area can be approximated by the measured enclosed loop area, i.e.,

(19) $$A_{eff}\approx A_{loop}\;$$

This probe-referenced area-scaling procedure avoids direct measurement of the relatively low induced voltage of small-area loops under strong transient electromagnetic excitation and maintains direct consistency between the experimental configuration and the geometric assumptions adopted in the numerical model. It, therefore, provides a reproducible measurement-to-response interface for subsequent ignition boundary evaluation.

### 3.3. Measured Magnetic Field Waveforms and Experimental Matrix

**Figure 5:**
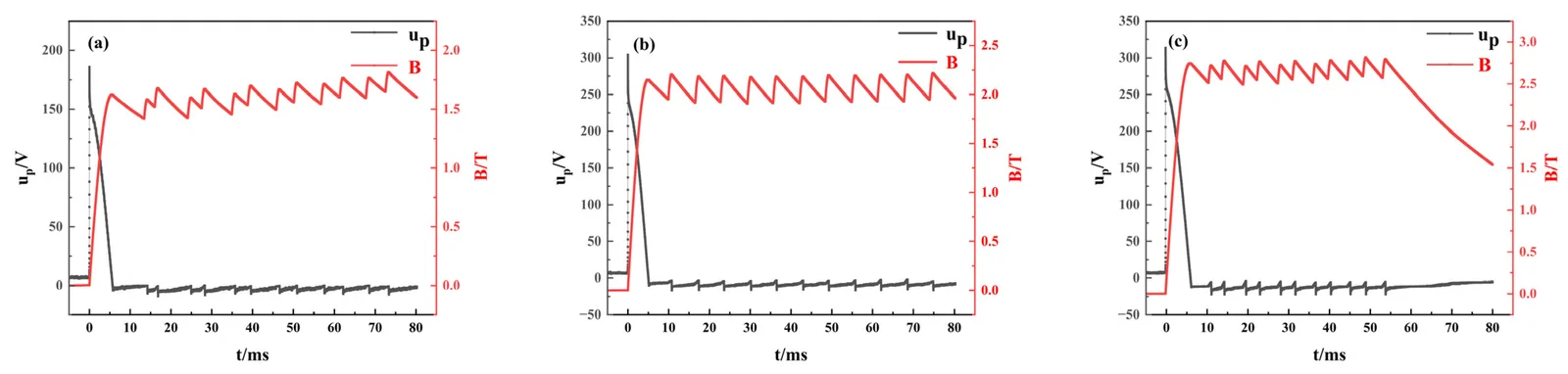
Three measured bench-top magnetic field waveforms and the corresponding induced-voltage curves: (**a**) Waveform I; (**b**) Waveform II; (**c**) Waveform III. The black and red curves represent u_p_ and B, respectively.

To evaluate the model-predicted ignition boundaries under different transient magnetic field inputs, three waveforms, denoted Waveforms I, II, and III, were experimentally generated and measured. As shown in Figure 5, each waveform comprised a finite rise stage, a short-duration high-field plateau, and a decay stage, while differing in peak magnetic flux density, maximum magnetic field variation rate, rise time, and effective pulse width. The measured waveforms were used instead of idealized trapezoids. The local magnetic field variation rate history, *dB*_exp_/*dt*, was obtained directly from the inductive-probe voltage, and *B*_exp_(*t*) was reconstructed by time integration. Numerical differentiation of *B*_exp_(*t*) waveform was, therefore, unnecessary. The characteristic parameters of the three waveforms are listed in Table 3.

For each waveform, firing tests were conducted using loops with different measured enclosed areas, with additional replicate tests near the critical ignition interval. The EED batch, initial resistance range, loop conductor, specimen position, and specimen orientation were held constant across all test conditions. Ignition or non-ignition was recorded after each test.

### 3.4. Ignition Responses and Critical Intervals for Different Loop Areas

For each magnetic field waveform, the measured enclosed loop area *A_loop_* was progressively increased, and the EED response was recorded. Replicate tests were performed near the critical interval to avoid interpreting device-to-device variability as a deterministic boundary. Measured loop areas were rounded to the nearest 1 mm^2^. Table 4 summarizes the ignition outcomes near the critical interval for all three waveforms in terms of the measured enclosed loop area.

As shown in Table 4, the critical loop area generally decreased as the maximum rate of change in magnetic flux density increased. For the quasi-trapezoidal waveforms tested here, this trend suggests that the peak induced voltage associated with *dB/dt*_max_ is an important factor affecting the critical ignition area. Waveforms II and III had similar *dB/dt*_max_ values and showed only a small difference in their measured critical-area intervals. Considering the experimental resolution and sample-to-sample variability, the two intervals were treated as approximately equivalent under the tested conditions.

### 3.5. Comparison of Simulated and Experimental Critical Boundaries

Table 5 compares the predicted and experimental critical-area intervals for the three measured waveforms. Because the equivalent loop radius was scanned in discrete increments of 1 mm, the numerical boundary is reported as an interval rather than a single value. Its lower bound is the largest radius that does not satisfy the ignition criterion, and its upper bound is the smallest radius that does. For an equivalent circular loop, the corresponding area is calculated as A = π*R*_loop_^2^.

Under Waveform I, the ignition criterion was not satisfied at an equivalent loop radius of 16 mm but was first satisfied at 17 mm, yielding a predicted critical-area interval of (804.2, 907.9] mm^2^. Experimentally, the largest measured enclosed area with no ignition was 772 mm^2^, whereas 824 mm^2^ was the smallest area at which ignition occurred in all replicate tests, giving an experimental interval of (772, 824] mm^2^. The two intervals overlapped within (804.2, 824] mm^2^, indicating that the predicted ignition boundary was consistent with the experimental results at the interval level.

For both Waveforms II and III, the predicted critical-area interval was (530.9, 615.8] mm^2^, whereas the experimental interval was (542, 583] mm^2^. The experimental interval was entirely contained within the predicted interval for both waveforms, indicating agreement between the predicted and experimental ignition boundaries at the interval level.

**Figure 6:**
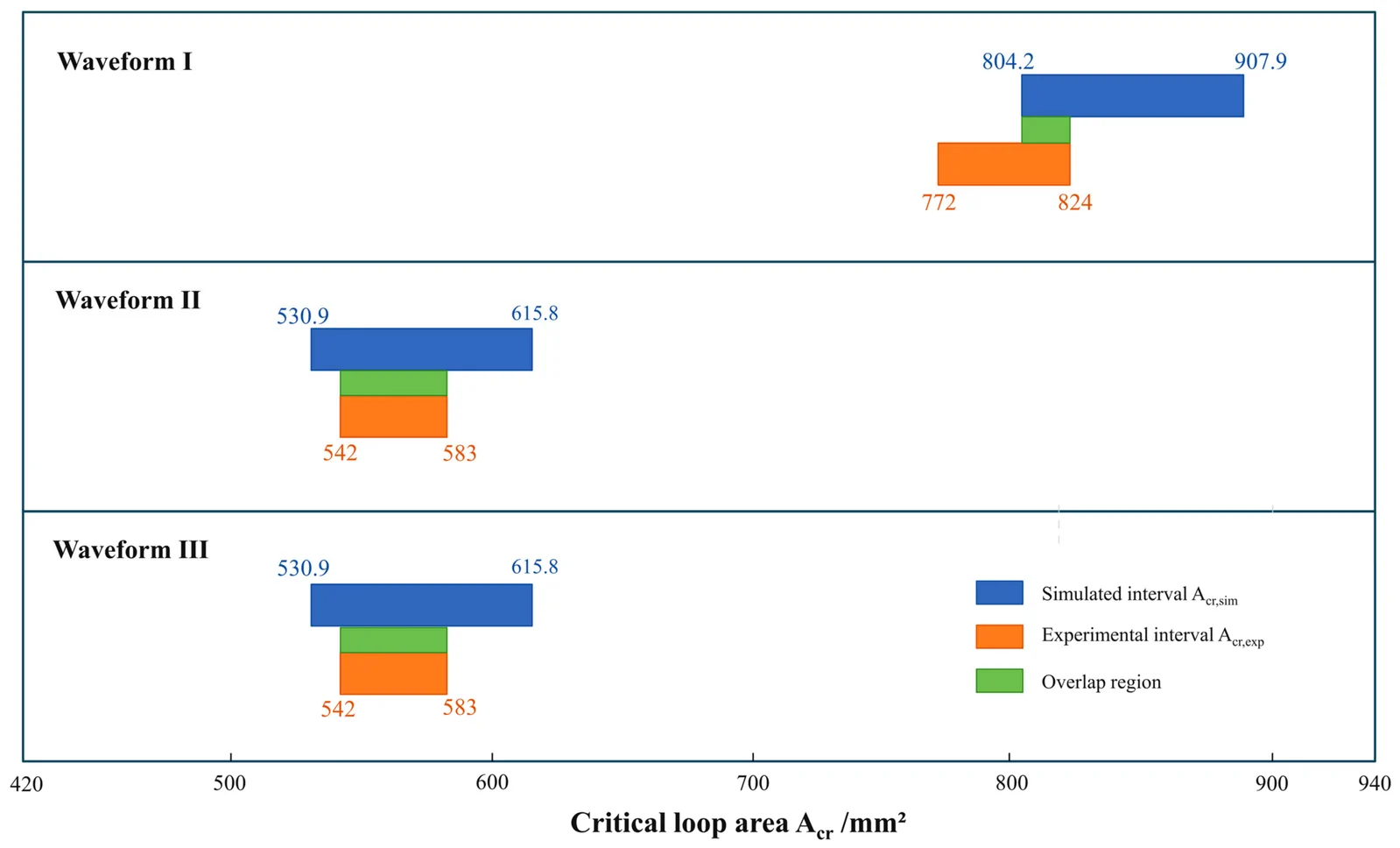
Comparison of simulated and experimental critical loop area intervals under three magnetic field waveforms.

As shown in Figure 6, the predicted critical intervals overlapped with the corresponding experimental intervals for all three waveforms. The predicted critical areas were slightly higher than the experimentally determined values. This upward deviation is consistent with the discrete radius increment used in the numerical scan. Because the equivalent loop radius was varied in increments of 1 mm, the numerical boundary could only be localized between the largest non-triggering radius and the smallest triggering radius. Converting these discrete radius bounds into loop areas, therefore, introduces a finite-resolution bias toward larger area values. Despite this discretization effect, the experimental and numerical results exhibited the same variation trend in critical area across the three waveforms. These results indicate that, within the tested ranges of magnetic field excitation, loop size, and EED characteristics, the proposed model captures both the magnitude and the variation trend of the critical ignition boundary with reasonable accuracy.

### 3.6. Temperature Field Characteristics of the Bridgewire–Charge System near the Critical Boundary

**Figure 7:**
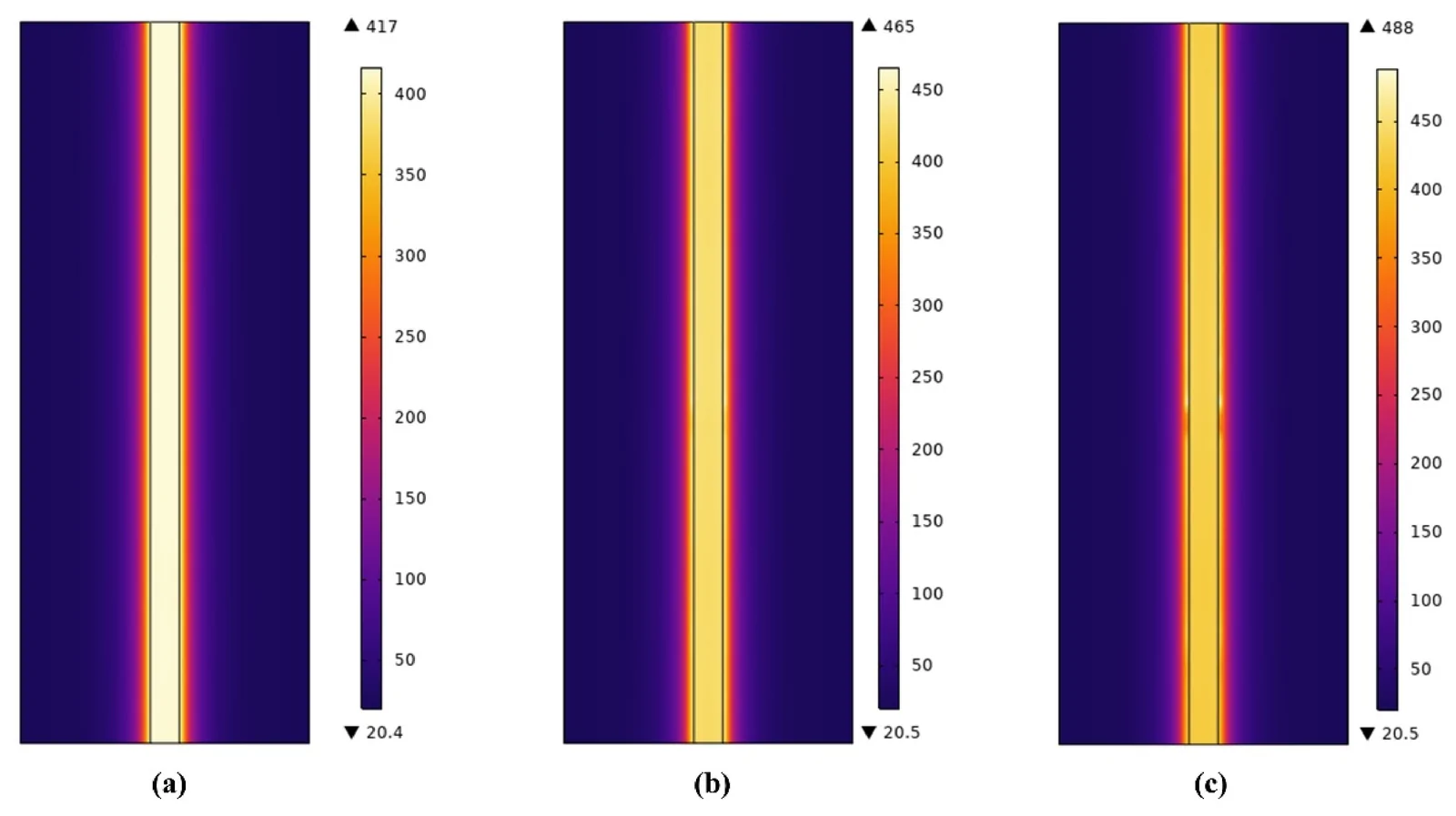
Temperature contours of the bridgewire–energetic material system near the critical boundary: (**a**) non-ignition condition with the loop radius below the critical radius; (**b**) near-critical condition with the loop radius approaching the critical radius; and (**c**) ignition risk condition with the loop radius above the critical radius.

To clarify the physical meaning of the critical loop radius, representative cases near the predicted boundary were examined. Figure 7 shows the temperature contours of the energetic charge under non-ignition, near-critical, and ignition risk conditions.

When *R*_loop_ was smaller than the critical radius, the maximum temperature remained concentrated near the bridgewire, and heat diffused outward into the surrounding energetic charge. No localized hot spot developed within the charge. The thermal response was, therefore, dominated by Joule heating in the bridgewire followed by thermal diffusion into the charge.

When *R*_loop_ approached the critical radius, the bridgewire–charge interface temperature increased markedly, and heat accumulation became more pronounced in the adjacent charge. Although the charge temperature still decreased with distance from the bridgewire, the temperature gradient became smaller and the high-temperature region near the interface broadened. No distinct internal temperature maximum had yet developed, but the local thermal state was approaching the instability threshold. This condition was regarded as a transitional state near the critical boundary.

When *R*_loop_ exceeded the critical radius, a local temperature maximum developed within the energetic charge. The high-temperature region was no longer confined to the bridgewire–charge interface but evolved into an internal hot spot. This behavior indicates that heat generation from exothermic decomposition had begun to exceed conductive heat loss, causing the dominant thermal response mechanism to shift from bridgewire-driven heating to self-accelerating heat release in the charge. The emergence of the internal temperature maximum corresponds to satisfaction of the ignition risk criterion, supporting the interpretation that the critical loop radius marks a transition in the dominant thermal response mechanism rather than an empirically prescribed temperature threshold.

**Figure 8:**
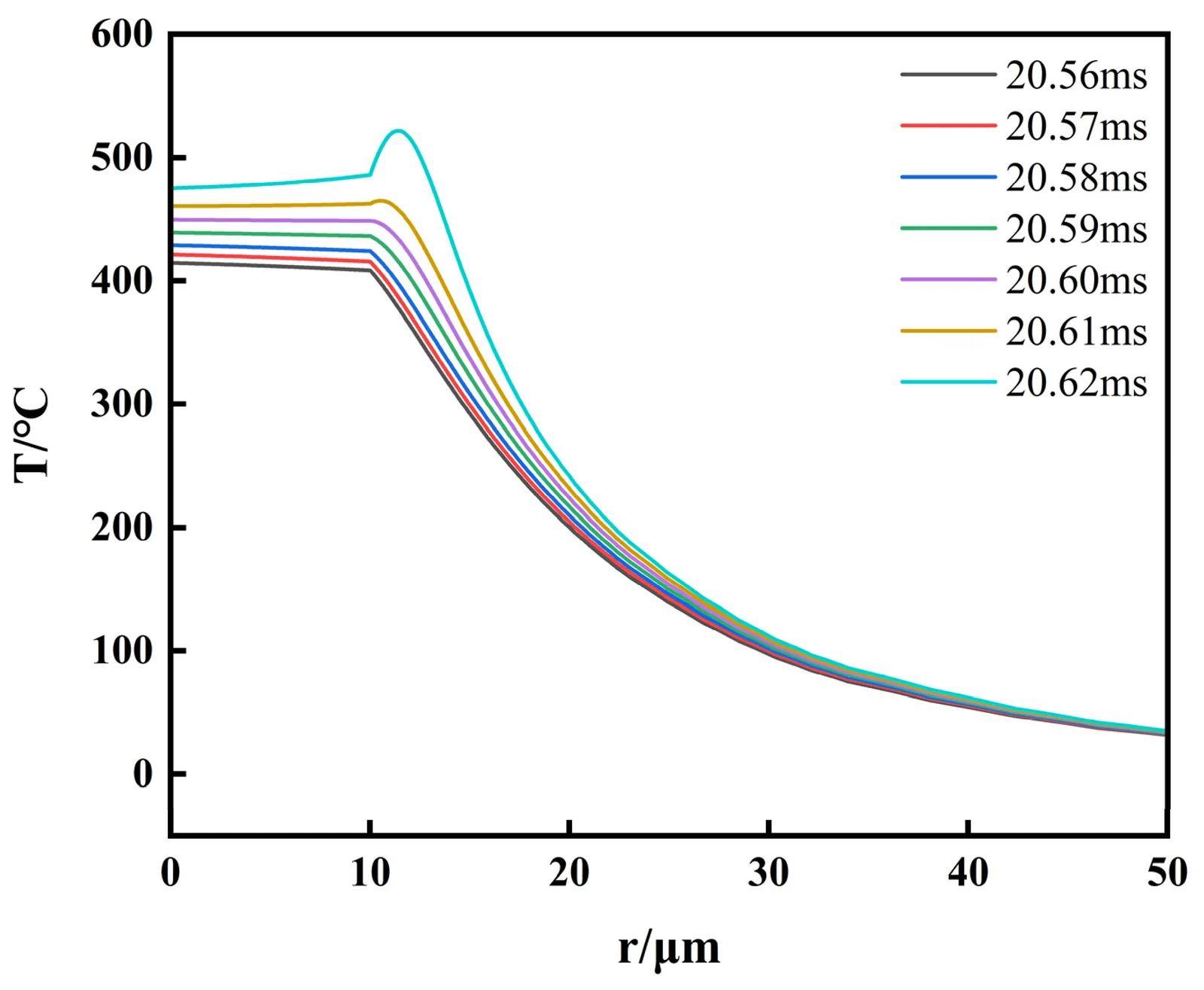
Local normal temperature profiles near the critical boundary.

Figure 8 presents the energetic–charge temperature profiles along the local normal direction for the corresponding cases. Under the non-ignition condition, the charge temperature decreased monotonically with increasing distance from the bridgewire. Under the near-critical condition, the magnitude of the temperature gradient decreased, and the profile gradually flattened toward a local plateau. Under the ignition risk condition, an internal temperature maximum developed, resulting in a distinctly non-monotonic temperature distribution. This result is consistent with the internal hot spot observed in Figure 7 and further supports the proposed criterion for identifying the transition from bridgewire-driven heating and heat diffusion to localized thermal instability.

### 3.7. Validation Summary and Discussion

The predicted and experimental critical intervals overlapped for all three bench-top magnetic field waveforms. For Waveform I, the overlap was (804.2, 824] mm^2^; for Waveforms II and III, the experimental interval of (542, 583] mm^2^ lay entirely within the predicted interval of (530.9, 615.8] mm^2^. Given the 1 mm radius step in the numerical scan and the finite experimental resolution, this agreement supports the predicted ignition boundary at the interval level. The thermal fields provide complementary evidence: increasing the loop radius changed the energetic–charge temperature profile from a monotonic distribution dominated by bridgewire-driven heating and heat diffusion to a locally non-monotonic distribution associated with thermal instability. Together, the boundary measurements and temperature field evolution support a consistent transition from bridgewire-driven heating and heat diffusion to local thermal instability. These results provide the basis for applying the projectile-fixed launch field *B*_launch_(*t*) in Section 4 to predict the critical loop radius under launch conditions.

The bench tests produced lower *dB/dt* values than those predicted under the simulated coilgun launch conditions. Section 3, therefore, evaluates the model’s boundary-prediction capability and thermal response behavior only within the experimentally accessible field range, whereas Section 4 extends the experimentally evaluated model to *B*_launch_(*t*). The resulting launch boundary should, therefore, be interpreted as a model-based prediction rather than a directly measured launch threshold.

## 4. Ignition Risk Prediction Under Launch Conditions

### 4.1. Projectile-Fixed Magnetic Field Exposure Characteristics During Launch

**Figure 9:**
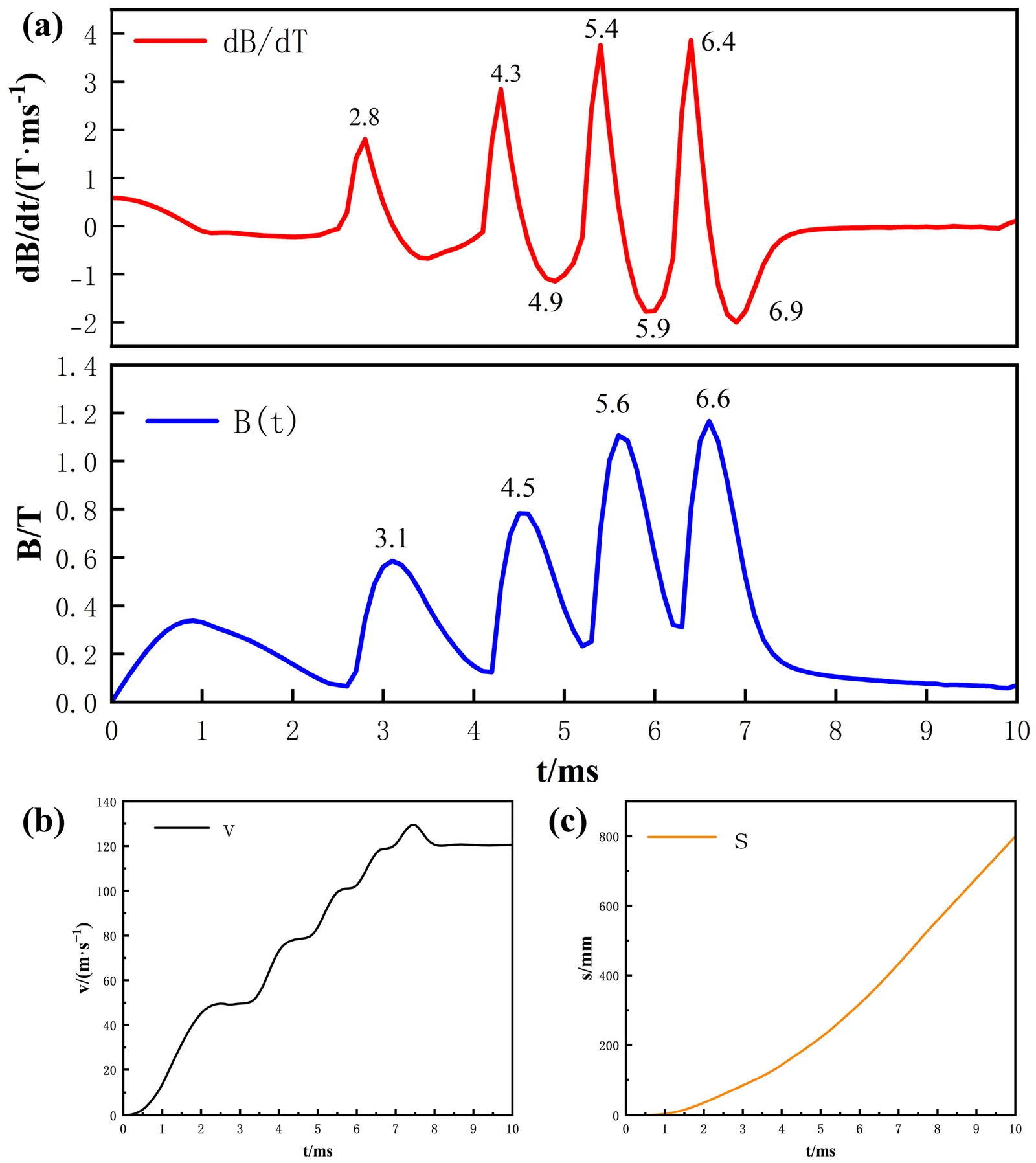
Launch kinematics and projectile-fixed magnetic field exposure of the EED installation region: (**a**) *B*_launch_(t) and *dB*_launch_/*dt*; (**b**) projectile velocity; (**c**) projectile displacement.

The multistage coilgun model described in Section 2.1 was solved using transient field–circuit coupling to obtain the velocity and displacement histories of the armature–projectile assembly, as shown in Figure 9b,c. Sequential discharge of the five drive coils produced a stepwise increase in velocity, with smaller velocity increments during the first two stages [36] and larger increments in the later stages. The effective acceleration interval lasted 7.4 ms, during which the assembly reached a peak velocity of 136.2 m/s. After the armature passed the optimal acceleration region, the capture effect [36] produced a decelerating electromagnetic force, reducing the velocity to a final value of 117.4 m/s. The final displacement was 0.8 m.

Figure 9 also shows the time histories of the local magnetic flux density, *B*_launch_ (*t*), and its rate of change, *dB*_launch_/*dt*, at the EED installation region. As the armature–projectile assembly passes the five drive coils, *B*_launch_ (*t*) exhibits five principal peaks that generally increase toward the later stages. Under the prescribed operating conditions, *B*_launch_ (*t*) reaches a maximum of 1.12 T at approximately 6.6 ms. The maximum positive and most negative values of *dB*_launch_/*dt* are 3.8 T/ms and −2.1 T/ms, respectively.

The results indicate that the inductive ignition risk of the bridgewire EED cannot be evaluated solely from the peak magnetic flux density. In the later launch stages, the higher projectile velocity increases the magnitude of the motion-induced term *v*(*t*)*∂B/∂x* in regions with strong spatial field gradients, producing larger *dB*_launch_/*dt* peaks near the fourth and fifth drive coils. The magnetic field history experienced by the EED, therefore, differs from that at a stationary spatial point. In the projectile-fixed frame, the exposure is a multipulse history jointly determined by coil discharge, projectile position and velocity, and the spatial field distribution. This trajectory-resolved *B*_launch_ (*t*) is used as the input for the launch condition ignition risk assessment.

Peak *dB*/*dt* primarily sets the peak induced voltage, but it does not determine ignition risk by itself. The thermal response also depends on the induced-current duration, loop impedance, bridgewire heat capacity, interfacial heat transfer, and heat dissipation between successive pulses. The launch condition assessment, therefore, considers both the instantaneous electromagnetic response and the cumulative thermal response.

### 4.2. Effects of Loop Size on Bridgewire–Charge Thermal Response and Ignition Risk

**Figure 10:**
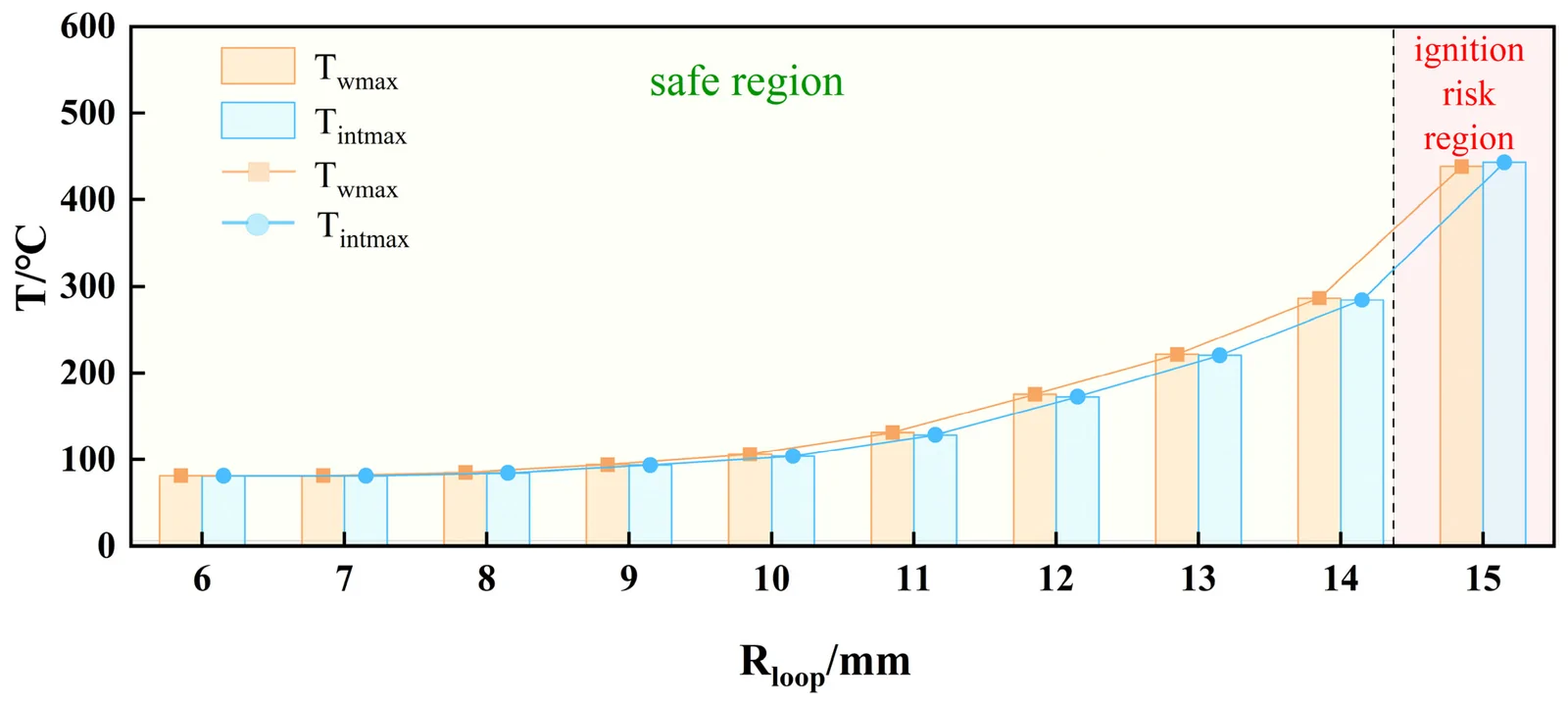
Thermal response evolution and safety status transition with increasing loop radius.

For the launch field calculation, the same *B*_launch_ (*t*) waveform was used while the equivalent loop radius *R*_loop_ was varied; all material properties, bridgewire dimensions, and energetic–charge parameters were kept unchanged. Varying *R*_loop_ represents changes in magnetic coupling caused by different internal wiring and return-path layouts. Table 6 lists the bridgewire–charge interfacial temperature and the maximum charge temperature for each radius, and Figure 10 shows the corresponding transition in thermal response. For small *R*_loop_, the temperature rise was limited, and the ignition risk criterion was not satisfied. As *R*_loop_ approached the critical range, later pulses deposited energy before heat from earlier pulses had fully dissipated, producing pronounced thermal accumulation. Once *R*_loop_ entered the ignition risk range, the ignition risk criterion was satisfied, indicating a transition to the ignition risk state.

### 4.3. Critical Loop Radius and Ignition Risk Boundary

The ignition risk criterion in Equation (13) is satisfied when *G*(*t*) > *G*_tol_. In the radius scan, the charge temperature profile remained monotonic along the local normal direction at *R*_loop_ = 14 mm. At *R*_loop_ = 15 mm, *G*(*t*) > *G*_tol_ was satisfied for the first time, indicating that local heat generation within the charge had begun to exceed conductive heat loss. With the 1 mm scan increment used here, the predicted critical loop radius, therefore, lies within the interval (14, 15] mm. The *R*_loop_ = 14 mm case is the largest tested radius that remained below the ignition risk criterion and is treated as a near-critical condition.

**Figure 11:**
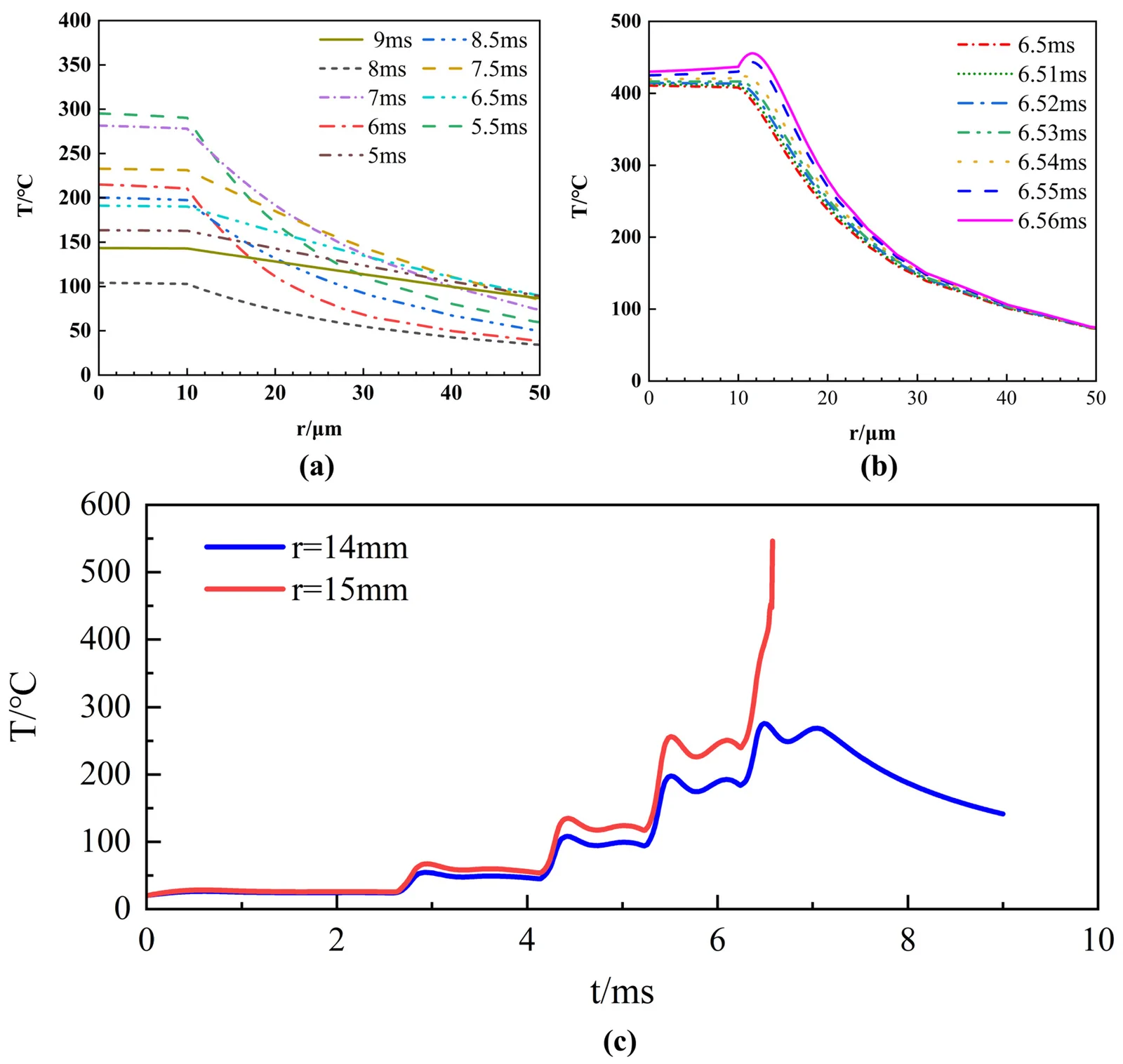
Effect of closed-loop radius on the bridgewire–charge thermal response and ignition risk criterion: (**a**) radial temperature distributions under the near-critical safe condition; (**b**) formation of a local temperature bulge under the ignition risk condition; (**c**) temperature evolution for *R*_loop_ = 14 and *R*_loop_ = 15.

Figure 11 further illustrates the thermal response evolution near the predicted critical loop radius. For *R*_loop_ = 14 mm, the temperature profiles shown in Figure 11a remain monotonically decreasing with increasing distance from the bridgewire throughout the selected time interval. Although successive magnetic field pulses produce repeated heating and thermal accumulation in the energetic charge, no internal temperature maximum develops, and the ignition risk criterion is not satisfied.

When *R*_loop_ is increased to 15 mm, the thermal response changes markedly, as shown in Figure 11b. With successive energy deposition, the temperature gradient near the bridgewire gradually decreases, and an internal temperature maximum eventually develops within the energetic charge. At this stage, the local normal temperature profile becomes non-monotonic, and the condition *G*(*t*) > *G*_tol_ is satisfied, indicating that local heat generation associated with exothermic decomposition has begun to exceed conductive heat loss.

The corresponding bridgewire–charge interfacial temperature histories are shown in Figure 11c. For both loop radii, the interfacial temperature increases in a stepwise manner as successive launch field pulses deposit additional energy before the residual heat from the preceding pulses has fully dissipated. For *R*_loop_ = 14 mm, the ignition risk criterion remains unsatisfied, and the temperature subsequently decreases after the final major heating stage. In contrast, for *R*_loop_ = 15 mm, the later pulses produce substantially stronger thermal accumulation, followed by a rapid temperature rise associated with the onset of local thermal instability. These results further support the predicted critical loop radius interval of (14, 15] mm.

### 4.4. Safety Margin, Engineering Constraints, and Model Applicability

The predicted critical loop radius range can be translated into a geometric safety margin. If the maximum equivalent closed-loop radius achievable within the projectile, *R*_loop,actual_, is sufficiently smaller than the lower bound of the predicted critical range, the wiring configuration has a positive geometric safety margin. Conversely, if *R*_loop,actual_ approaches or enters the critical range, the geometric margin decreases and the configuration approaches the predicted ignition risk region. Magnetic flux coupling can be reduced by decreasing the enclosed loop area, routing the outgoing and return conductors closer together, placing the return conductor close to the lead wires, or adjusting the loop orientation. Where appropriate, increasing the loop impedance can further reduce the induced current and associated Joule heating.

In this study, the geometric safety margin is defined as

(20) $$M_{R}=\frac{R_{\mathrm{loop},\mathrm{cr}}-R_{\mathrm{loop},\mathrm{actual}}}{R_{\mathrm{loop},\mathrm{cr}}}$$

Here, *M_R_* > 0 denotes a positive geometric margin, *M_R_* = 0 indicates that the equivalent loop size has reached the adopted limit, and *M_R_* < 0 indicates that the limit has been exceeded. For a conservative margin calculation, *R*_loop,cr_ is set to 14 mm, the largest radius that remained below the ignition risk criterion in the discrete scan. The resulting margins are approximately 0.43, 0.29, 0.14, and 0.07 for *R*_loop,actual_ = 8, 10, 12, and 13 mm, respectively. For *R*_loop,actual_ ≥ 15 mm, M*_R_* is negative, and the configuration lies beyond the adopted limit.

The present model describes the coupled electromagnetic–thermal response up to the onset of the ignition risk condition; post-ignition combustion, gas generation, pressure evolution, structural failure, and fragment ejection are beyond its scope. Bench tests evaluated whether the model could distinguish ignition from non-ignition and capture the critical boundary under measured magnetic field histories. Launch condition risk was then predicted by applying the experimentally evaluated model to *B*_launch_(*t*) in the projectile-fixed frame. For other coilgun configurations, EED orientations, or internal wiring layouts, *B*_launch_(*t*) must be re-extracted, while *A*_eff_ and the loop impedance must be re-evaluated accordingly.

## 5. Conclusions

A measurement-based framework was developed to evaluate the inductive ignition risk of projectile-borne bridgewire EEDs under transient magnetic field exposure by linking magnetic field excitation, closed-loop electrical response, and bridgewire–charge electromagnetic–thermal behavior. The main conclusions are as follows.

(1) Under three experimentally measured transient magnetic field waveforms, the predicted ignition boundaries overlapped with the experimentally determined critical intervals. For Waveform I, the experimental and predicted critical area intervals were (772, 824] and (804.2, 907.9] mm^2^, respectively. For Waveforms II and III, the experimental interval (542, 583] mm^2^ lay entirely within the predicted interval (530.9, 615.8] mm^2^. The consistent interval-level agreement across the three transient field inputs indicates that the model captures the variation in the ignition boundary under different transient magnetic field excitations.
(2) Applying the experimentally evaluated model to the projectile-fixed magnetic field history of the five-stage coilgun yielded a predicted critical equivalent loop radius interval of (14, 15] mm. The ignition risk criterion was not satisfied at *R*_loop_ = 14 mm but was satisfied at *R*_loop_ = 15 mm. Ignition risk, therefore, reflects the full multi-pulse excitation history and associated thermal accumulation, not peak magnetic flux density or peak *dB/dt* alone.
(3) The framework links measurable transient magnetic fields to closed-loop electrical excitation and device-level ignition risk boundaries. It, therefore, provides a quantitative basis for establishing design constraints on loop size, conductor routing, and installation orientation in the electromagnetic safety design of projectile-borne EEDs.

The present model is limited to the electromagnetic–thermal response up to the onset of the ignition risk condition and does not describe post-ignition combustion, gas generation, pressure evolution, or structural failure. The bench tests also covered lower *dB/dt* levels than those predicted under the simulated coilgun launch conditions. The predicted (14, 15] mm launch boundary should, therefore, be interpreted as a model-based extrapolation supported by bench-scale boundary validation, rather than as a directly measured launch threshold.

## Figures and Tables

**Table 1:** Model parameters of the multistage coilgun.

Category	Parameter	Value	Description
Drive coil	Number of stages	5	Five-stage synchronous induction coil
	Turns per coil	100	Identical for all stages
	Inner diameter/mm	49	Clearance reserved relative to the armature outer diameter
	Outer diameter/mm	79	Equivalent model dimension
	Length of each stage/mm	90	Axial length
	Interstage spacing/mm	20	Spacing between adjacent coils
Projectile assembly	Mass/kg	3.58	Equivalent mass including the armature and projectile body
Armature	Length/mm	60	Annular solid armature
	Inner diameter/mm	16	Equivalent inner bore
	Outer diameter/mm	46	Matched with the coil inner diameter

**Table 2:** Material properties of the bridgewire 6J20 and charge.

Properties	Bridgewire 6J20 [34]	Energetic Charge [34]
*r*/μm	10	-
*λ*/W·(m·K)^−1^	-	0.88
*ρ*/kg·m^−3^	8300	1468
*c*/J·(kg·K)^−1^	460	1023
*σ*/S·m^−1^	9.26 × 10^5^	-
*α*/K^−1^	1.5 × 10^−4^	-
*Q*/J·kg^−1^	-	6.90 × 10^6^
*E*/R*/K	-	9600
*A*/s^−1^	-	1 × 10^9^

Note: *r* is the radius of the bridgewire. *λ* is the thermal conductivity of the reagent. *ρ* is the density. *c* is the specific heat capacity. *σ* is the bridgewire conductivity. *α* is the temperature coefficient of the bridgewire resistance. *Q* is the combustion heat per unit mass of the reagent. *E* is the activation energy of the reagent. R* is the universal gas constant. *A* is the frequency factor.

**Table 3:** Characteristic parameters of the three measured magnetic field waveforms.

Waveform	*B*_max_/T	*dB*/*dt*_max_/T·ms^−1^	Rise Time/ms
I	1.28	3.21	5.2
II	2.24	4.57	5.1
III	2.73	4.78	4.6

**Table 4:** EED responses at different effective areas under the three measured waveforms.

Waveform	No.	*A_loop_*/mm^2^							
		445	507	542	583	669	706	772	824
I	1	-	×	-	-	-	-	-	-
	2	-	-	×	-	-	-	-	-
	3	-	-	-	×	-	-	-	-
	4	-	-	-	-	×	-	-	-
	5	-	-	-	-	-	×	-	-
	6	-	-	-	-	-	-	×	-
	7	-	-	-	-	-	-	×	-
	8	-	-	-	-	-	-	×	-
	9	-	-	-	-	-	-	-	√
	10	-	-	-	-	-	-	-	√
	11	-	-	-	-	-	-	-	√
II	12	×	-	-	-	-	-	-	-
	13	-	×	-	-	-	-	-	-
	14	-	-	×	-	-	-	-	-
	15	-	-	×	-	-	-	-	-
	16	-	-	×	-	-	-	-	-
	17	-	-	-	√	-	-	-	-
	18	-	-	-	√	-	-	-	-
	19	-	-	-	√	-	-	-	-
III	20	×	-	-	-	-	-	-	-
	21	-	×	-	-	-	-	-	-
	22	-	-	×	-	-	-	-	-
	23	-	-	×	-	-	-	-	-
	24	-	-	×	-	-	-	-	-
	25	-	-	-	√	-	-	-	-
	26	-	-	-	√	-	-	-	-
	27	-	-	-	√	-	-	-	-

Note: √ denotes ignition; × denotes no ignition.

**Table 5:** Comparison of simulated and experimental critical-area intervals for three waveforms.

Waveform	*R*_cr,sim_/mm	*A*_cr,sim_/mm^2^	*A*_cr,exp_/mm^2^	Overlap
I	(16, 17]	(804.2, 907.9]	(772, 824]	yes
II	(13, 14]	(530.9, 615.8]	(542, 583]	yes
III	(13, 14]	(530.9, 615.8]	(542, 583]	yes

Note: *R*_cr,sim_ denotes the simulated critical radius interval; *A*_cr,sim_ and *A*_cr,exp_ denote the simulated and experimental critical-area intervals, respectively.

**Table 6:** Ignition risk prediction results under different closed-loop radii.

*R*_loop_/mm	*A*_eff_/mm^2^	*T_w_*_max_/°C	*T_int_*_max_/°C	*G*(*t*) − *G_tol_*	Safety Status
6	113.1	81.6	81.3	<0	safe
7	153.9	81.6	81.3	<0	safe
8	201.1	85.2	84.7	<0	safe
9	254.5	94.2	93.5	<0	safe
10	314.2	106	103.9	<0	safe
11	380.1	131.1	128.4	<0	safe
12	452.4	175.3	172.6	<0	safe
13	530.9	221.5	220.3	<0	safe
14	615.8	286.4	284.3	<0	safe
15	706.9	438.4	443.2	>0	Ignition risk

## Data Availability

The data presented in this study are available from the corresponding author upon reasonable request.
